# Individual and Contextual Morality: How Educators in Oppositional and Permissive Communities Use Culturally Responsive Practices

**DOI:** 10.3390/bs15040446

**Published:** 2025-03-31

**Authors:** Kate M. Morman, Cong Wang, Laura M. Brady, Stephanie A. Fryberg

**Affiliations:** 1 Department of Psychology, Northwestern University, Evanston, IL 60208, USA; 2 Education Systems and Policy Program Area, American Institutes for Research, Chicago, IL 60606, USA

**Keywords:** culturally responsive practices, diversity ideologies, moral beliefs in education, equity-focused teaching in political contexts

## Abstract

Instructional practices that attend to students’ cultural motivations and strengths can play an important role in mitigating educational inequities. However, educators increasingly experience backlash for efforts to address educational inequities, raising moral questions about how educators should engage students. Through a national study, we explored how educators’ likelihood of implementing culturally responsive practices (CRPs) (i.e., practices focused on affirming students’ cultural backgrounds) varied according to educators’ individual moral frameworks (i.e., multicultural and colorblind diversity ideologies) and the contextual moral frameworks they encountered among their administrators (i.e., support for educational equity work) and local communities (i.e., DEI sentiment). When their communities were permissive of DEI, teachers who strongly endorsed multiculturalism implemented CRPs frequently, regardless of their administrators’ support for equity work. In DEI-opposed communities, however, pro-multiculturalism educators only implemented CRPs frequently when their administrators supported equity work. In contrast, regardless of community-level DEI sentiment, CRP implementation among educators with weaker endorsement of multiculturalism depended upon administrators’ support for equity work. Results suggest that educators with less well-defined individual moral frameworks about diversity rely upon contextual frameworks to determine their practices, while those with more codified moral frameworks rely upon contextual frameworks primarily when their individual moral frameworks conflict with their community’s.

## 1. Introduction

Racial and socioeconomic achievement gaps are a bleak and enduring reality in U.S. education. Compared to White students and students from higher-income families, many students from marginalized backgrounds^1^ experience lower quality educational opportunities and worse academic outcomes (e.g., GPA, test scores, educational attainment) (Musu-Gillette et al., 2016). While racial and socioeconomic disparities have long plagued the educational system, the debate about alleviating these disparities has reached a new level of intensity. At the local level, many schools and teachers have faced backlash about what and how they teach, with parent organizations calling for bans on books and discussions of equity issues (Pollock, 2023). At the state level, more than 100 bills were introduced in 2024 to ban diversity, equity, and inclusion (DEI) efforts in K-12 and higher education (Alexander et al., 2024). At the federal level, Republican legislators introduced the Dismantle DEI Act of 2024, which would eliminate federal funding for DEI-related activities, programs, and offices across all areas of government, including education (Dismantle DEI Act of 2024, 2024).

Opposition toward efforts to create equitable and inclusive educational systems has grown, despite decades of research demonstrating that these efforts can enhance students’ learning experiences and outcomes (e.g., Banks, 2008; Brady et al., 2024; Sleeter, 2011; Stephens et al., 2012, 2014). As schools across the country grapple with widening racial/ethnic and socioeconomic achievement gaps, the implementation of practices that have the potential to mitigate educational inequities is perhaps even more pertinent. We contend that educators’ decisions about whether and how to use such practices likely depends upon both educators’ individual moral frameworks and the contextual moral frameworks they encounter among their administrators and local communities. Through a national study of K-12 teachers, we explored teachers’ likelihood of implementing a particular set of equity-supportive teaching practices, culturally responsive teaching practices (CRPs; practices that validate students’ cultural backgrounds and communicate that these backgrounds are welcome at school). We examined how teachers’ implementation of CRPs varies according to their personal moral frameworks about the role of diversity in education (i.e., diversity ideologies), the support for educational equity work that they perceive among their administrators, and the extent to which their surrounding community is opposed to DEI efforts.

### 1.1. Diversity Ideologies Provide Individual Moral Frameworks

Diversity ideologies are widely shared beliefs about the right way to recognize and respond to cultural diversity (Gündemir et al., 2019; Plaut, 2002), providing guidance for how individuals *should* behave in relation to people with different backgrounds and experiences (Wu & Apfelbaum, 2024). In this way, diversity ideologies can function as moral frameworks that people use to identify the right course of action in contexts that include people from different cultural backgrounds, particularly when there is concern about the fairness of individuals’ treatment or outcomes in these contexts (Apfelbaum et al., 2016; Ely & Thomas, 2001).

The two prevailing diversity ideologies in the U.S. center on colorblindness and multiculturalism (Wu & Apfelbaum, 2024). Colorblind diversity ideologies convey that the morally “right” way to approach diversity is to avoid drawing attention to differences or treating people differently based on their cultural, racial/ethnic, social class, and other consequential identities (Knowles et al., 2009; Leslie & Flynn, 2022). Instead, people who endorse colorblind ideologies believe that the focus should be on seeing others as individuals and ensuring that all individuals have access to the same opportunities and resources. Conversely, multiculturalism diversity ideologies convey that the morally “right” way to approach diversity is to celebrate differences among people as strengths (Plaut et al., 2011). Individuals who endorse this ideology may be more likely to acknowledge that people have different needs and motivations depending on their cultural backgrounds and therefore should be supported in different ways (Apfelbaum et al., 2016; Plaut et al., 2018).

People vary in the extent to which they endorse colorblind and multicultural diversity ideologies and use these ideologies to guide their actions. In educational settings, diversity ideologies may predispose teachers to adopt particular instructional practices that are more (or less) attuned to students’ cultural backgrounds, particularly CRPs. In contrast to normative instructional practices within the U.S., which do not consistently attend to students’ cultural backgrounds, CRPs derive from a body of literature that situates culture as a key aspect of learning (Gay, 2015; Paris, 2012; Paris & Alim, 2014). CRPs aim to improve students’ engagement and the quality of their educational experiences by (a) explicitly affirming students’ distinct cultural experiences, perspectives, and knowledge; (b) fostering perspective-taking; and (c) maintaining rigorous expectations for all students (Aronson & Laughter, 2016; Ladson-Billings, 1995). Given the centrality of students’ cultural backgrounds to CRPs, teachers who endorse multicultural diversity ideologies may find these practices to be more morally aligned with their beliefs compared to teachers who endorse colorblind ideologies. Indeed, teachers who endorse colorblind ideologies often favor practices that downplay or ignore cultural differences in favor of group cohesion, merit, and/or individuality (Aragón et al., 2017; Civitillo et al., 2019; Vittrup, 2016). Conversely, teachers who endorse multicultural ideologies often favor practices that celebrate and value cultural differences (Birnbaum et al., 2021; Brady et al., 2024).

### 1.2. Contextual Moral Frameworks Shape Individuals’ Experiences and Behaviors

While individuals develop their own understandings of the morally right ways to approach issues of diversity, organizations (e.g., schools, districts, and state boards of education) also convey support for multicultural and colorblind diversity ideologies in both subtle and explicit ways (Markus et al., 2000; Murphy et al., 2018; Murphy & Walton, 2013). For example, many schools and districts tout policies or statements that affirm their commitment to treating students, families, and staff equitably (Pew Research Center, 2023). Some of these statements center colorblindness, focusing on equitable treatment regardless of individuals’ racial/ethnic or other identities (Welton et al., 2015), while others center multiculturalism, focusing on celebrating their communities’ diverse cultural backgrounds and deriving strength from this diversity (e.g., Peetz & Najarro, 2024). Other districts say little to nothing about equitable treatment or outcomes, conveying that these topics are not focal to their educational mission or approach. These messages have implications for how educators behave in relation to culturally diverse students and families.

What is known about how schools enact diversity ideologies has been learned by studying school administrators, whose actions work to interpret policies into everyday norms and routines (Spillane et al., 2002). Within the web of administrator actions, we can examine how administrator support conveys a contextual morality about the “right” ways to approach diversity within schools (Peurach et al., 2019). Administrator support for educational equity ranges from everyday social interactions to strategically aligned curricula and evaluation materials, which can influence teachers’ beliefs and behaviors (Blaushild, 2023). For example, in schools where student disadvantages are positioned as individual choice rather than systemic, the everyday processes and policies that administrators develop provide a common framework to explain these inequalities among students (Cobb, 2017). These diversity frameworks can legitimize inaction, positioning unresponsive school policies and teacher practices as appropriate responses to student differences (Liou et al., 2019). On the other hand, explicit and active administrator support for educational equity can also extend a positive influence on teachers and increase the adoption of inclusive practice (Hagenaars et al., 2023). While these studies offer in-depth examinations regarding the role that administrator support plays in enacting contextual morality through everyday routines and norms, large-scale evidence of diversity ideologies embedded within schools is nascent and warrants further investigation.

### 1.3. DEI Opposition Shapes Individual Awareness and Practices

Beyond school- or district-level support for educational equity, teachers are aware of the broad political climate within their communities, and this awareness may influence their decisions about what and how to teach (McAvoy & Hess, 2013). Within education research, it is often taken for granted that teachers have some awareness of their community’s political climates, making precise differences between teachers’ moral frameworks and the influence of local contexts difficult to trace. For example, national surveys following the 2016 US presidential election demonstrated that teachers had an increased awareness of their community’s political climates and felt that these influenced their practice implementation (Dunn et al., 2019). However, teachers were not asked about the degree to which their communities were liberal or conservative; teachers’ awareness of their community’s political climate created enough concern to influence their practices. Since 2021, community political climates across the US have seen an increase in state-level legislation restricting teachers’ discussion of issues related to race and gender (Alexander et al., 2024). These laws indicate a broad opposition to a focus on equity within education, rooted in moral frameworks that convey that focusing on equity is divisive, unfair, or even harmful to certain students and families. National surveys indicate that teachers in states with these laws experience more restrictions on their practices than teachers in states without these laws (Woo et al., 2022). Practice restrictions were particularly prevalent among politically conservative communities compared to politically liberal communities in states with anti-DEI legislation (Woo et al., 2023). Recent qualitative research established that educators within anti-DEI communities are using contextual morality cues from their administrators and communities to make decisions about their practice implementation (Kelly et al., 2024; Pollock, 2023). However, research has yet to contend with the moderating influence that teachers’ more and less codified moral frameworks may play on the contextual-derived moral frameworks they encounter within their schools and communities.

### 1.4. Current Research

Teachers increasingly find themselves at a crossroads when it comes to engaging with educational equity issues in the classroom. Each year, a growing proportion of students from marginalized racial/ethnic and lower-income backgrounds enter the U.S. educational system. At an aggregate level, the outlook on their educational opportunities and likely outcomes is bleak relative to the outlook for their White and higher-income peers. One of the most promising strategies for mitigating these disparities is through implementation of practices such as CRPs, which are attuned to student’s cultural motivations and strengths. However, these practices may or may not align with teachers’ own moral frameworks for navigating diversity or with the frameworks they encounter among their administrators and in their local communities. While educators with strong individual moral frameworks regarding diversity may use these frameworks to guide their decisions about implementing CRPs, these decisions may also depend upon the support for or opposition to DEI that educators perceive among their school and district leaders and in their community more broadly.

Drawing on past research, we anticipate that educators who more strongly endorse multicultural diversity ideologies will be more likely to implement CRPs than those with weaker endorsements, as CRPs align with their individual moral frameworks for understanding diversity. However, while educators are moral agents with their own unique ways of making sense of and responding to diversity, they are also individuals embedded within school and community contexts that convey moral understandings that may conflict with their own. Little research has explored how educators navigate the contextual moral frameworks regarding educational equity that are prevalent at this moment in time. However, recent interviews with teachers in contexts opposed to educational equity efforts suggest that administrators’ responses may influence teachers’ practice decisions (Kelly et al., 2024; Pollock, 2023). When administrators actively protected educators’ ability to discuss equity issues in the face of gag orders and other state-level anti-DEI legislation, educators reported feeling more empowered to do so. However, when administrators either took little protective action or actively participated in anti-DEI efforts, educators reported feeling silenced or subdued and therefore less likely to discuss DEI (Pollock, 2023). Another study found through focus group interviews that educators in a state with anti-DEI legislation often cited their administrator’s opposition to this legislation when discussing how they continued to prioritize equity in spite of the legislation (Kelly et al., 2024).

While previous findings suggest that administrator support may play a critical role in shaping educators’ implementation of practices that support educational equity, particularly in anti-DEI contexts, we do not know the extent to which these findings depend upon educators’ moral frameworks for thinking about diversity. Prior studies leveraged convenience samples of educators who participated in justice-oriented teacher preparation programs or those who were particularly motivated to discuss issues related to educational equity and growing opposition to DEI in their communities. These educators may have been relatively homogenous in terms of their moral frameworks about diversity. It remains to be seen how educators with differing or less codified frameworks about diversity make decisions regarding equity-supportive practices. Through a national study of K-12 educators, we investigated these questions, exploring how teachers navigate their roles as moral agents within an education system increasingly shaped by ideological battles over diversity and equity.

## 2. Materials and Methods

### 2.1. Participants

Participants included K-12 educators from all U.S. states, districts, and territories, except Wyoming. We worked with Qualtrics Research Panels to recruit a national sample of K-12 teachers and administrators with a goal that at least 20% of participants identified as educators of color to ensure parity with national educator workforce demographics. The sample included certified teachers, specialists, or paraprofessionals (*N* = 1097). Most participants self-identified as female (81%) and White (76%). The majority (80%) held a bachelor’s degree. Approximately 48% taught in suburban schools, 30% in urban schools, and 21% in rural schools. Half of the participants (51%) taught general education, while 19% taught Special Education. Participants on average had 15 years of teaching experience, excluding student teaching.

### 2.2. Measures

#### 2.2.1. Diversity Beliefs

We operationalized diversity beliefs via teachers’ endorsement of two prevalent diversity ideologies in the U.S.: multiculturalism and colorblindness. Five items assessed endorsement of multiculturalism (α = 0.86; Mean = 4.63; SD = 0.96), such as “Teachers should use instructional materials that reflect the racial and ethnic diversity of their students”. Four items assessed endorsement of colorblindness (α = 0.76; Mean = 3.23; SD = 1.17), including items like “Given the diversity of students, educators should encourage racial and ethnic minorities to adapt to mainstream expectations of society”. Participants responded to all items using a 6-point scale (1—Strongly Disagree to 6—Strongly Agree). Each of these scales was used previously in educational research contexts (Brady et al., 2024).

#### 2.2.2. Culturally Responsive Practices

Implementation of CRPs was assessed using five items adapted from the Culturally Responsive Teaching Practices scale (Abacioglu et al., 2020). These items measured the frequency with which educators consider and integrate students’ backgrounds into classroom instruction and activities (α = 0.84; Mean = 4.02; SD = 1.21). We asked teachers: If someone were to observe your instruction, how often would they see you use each of the following practices? Questions included: “Use the cultural background of my students to make learning meaningful”. Teachers responded using a 6-point scale (1—Never to 6—A few times a day).

#### 2.2.3. Administrator Support for Educational Equity

Administrator Support for Educational Equity was assessed at both district and school levels. Two items assessed educators’ perceptions of their school administrators’ support for equity (α = 0.79; Mean = 4.56; SD = 1.12), such as “My school prioritizes equity work”. Two items assessed educators’ perceptions of their district administrators’ support for equity (α = 0.81; Mean = 4.25; SD = 1.21), including items like “My district prioritizes equity work”.

#### 2.2.4. Political Climate for DEI

We created a proxy measure using participants’ state and zip codes to assess the political climate regarding DEI. First, we consulted a public database that tracks local, state, and federal efforts to restrict critical race theory, diversity, equity, inclusion, antiracism, and anti-sexism efforts (Alexander et al., 2024). States with active anti-DEI legislation (i.e., regulation restricting K-12 content/activities on diversity and inclusion, including race/ethnicity and culture) were coded as 1, while states without such laws were coded as 0. Second, we used participants’ self-reported zip codes to determine community-level support for the Republican candidate in the 2020 general election based on data from the Presidential Precinct Map 2020 (Watkins, 2024). Precincts, where a majority of voters voted for the Republican candidate, were coded as 1, and precincts, where the majority voted for a candidate other than the Republican candidate, were coded as 0. Using these variables, we created a composite measure representing the political climate for DEI. Teachers in states with anti-DEI legislation and precincts where the majority voted for the Republican candidate in 2020 were coded as teaching in a “DEI-opposed” community (*N* = 252). In contrast, teachers in states without anti-DEI legislation and in precincts where the majority did not vote for the Republican candidate in 2020 were coded as teaching in a “DEI-permissive” community (*N* = 333). These variables are aligned with proxy variables for political climate used in other studies (Pollock, 2023; Woo et al., 2023).

### 2.3. Analytical Strategy

To examine how educators’ personal moral and contextually derived frameworks for educational equity predicted their implementation of CRPs in contexts that were more (or less) opposed to DEI, we ran a regression analysis with a 3-way interaction between educators’ diversity beliefs, administrator support for educational equity, and community opposition or permissiveness for DEI. We first ran a latent profile analysis with two types of diversity ideologies (i.e., multiculturalism, and colorblindness) to identify groups of teachers with distinct moral beliefs regarding diversity in education. The fit indices, including AIC, AWE, BIC, CLC, and KIC (Akogul & Erisoglu, 2017), were considered to determine the optimal classification. After identifying the groupings of teachers, we used linear regression analyses to examine how teachers’ moral beliefs about diversity, administrator support, and political climate interact to shape their use of CRPs. Teachers’ race and gender were included as covariates. When significant interactions among the variables were identified, we conducted simple slope analyses to further examine how teachers respond under varying conditions. All statistical analyses were conducted using R version 4.4.1 (R Core Team, 2024).

## 3. Results

### 3.1. Educators with Varying Moral Beliefs About Diversity

**Figure 1:**
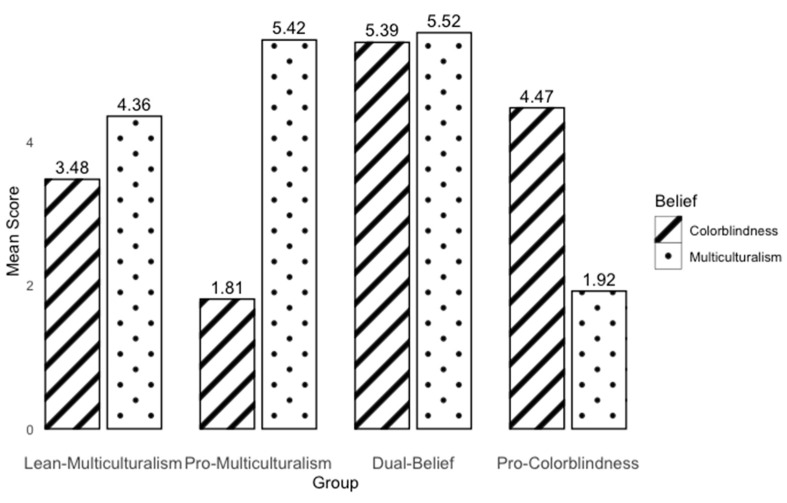
Mean multiculturalism and colorblindness scores for the four identified teacher groups.

To identify educators with varying moral beliefs about diversity, we conducted a latent profile analysis using teachers’ composite scores for multiculturalism and colorblindness. The result suggested that the best solution was a model with four groups (AIC = 5967.27; BIC = 6062.28; Entropy = 0.84). **Figure 1** presents the mean scores of multiculturalism and colorblindness for each group.

The largest group, which we refer to as “lean-multiculturalism” (*N* = 697; 64% of the whole sample), consisted of teachers who endorsed multiculturalism beliefs at a moderate level but lacked a clear stance on colorblindness. Specifically, their mean multiculturalism score (M_multiculturalism_ = 4.36; SD_multiculturalism_ = 0.71) suggested they somewhat agreed or agreed with multiculturalism, while their mean colorblindness score (M_colorblindness_ = 3.48; SD_colorblindness_ = 0.71) reflected attitudes ranging from somewhat disagree to somewhat agree. Repeated measure analysis indicated that their mean score for multiculturalism was significantly higher than for colorblindness (*p* < 0.001).

The second largest group, which we refer to as “pro-multiculturalism” (*N* = 278; 25% of the whole sample) exhibited strong endorsement of multiculturalism beliefs and low endorsement of colorblindness. They generally believe that educators should actively consider students’ backgrounds and incorporate them into classroom teaching and learning. Repeated measure analysis indicated that the mean score for multiculturalism was significantly higher than for colorblindness (M_multiculturalism_ = 5.42; SD_multiculturalism_ = 0.44; M_colorblindness_ = 1.81; SD _colorblindness_ = 0.47; *p* < 0.001).

Two smaller groups also emerged from the analysis, but they will not be considered beyond the initial description due to their limited sample sizes. The “dual-belief” group (*N* = 82; 7% of the whole sample) included teachers who strongly endorsed both multiculturalism and colorblindness, though their mean score for multiculturalism was significantly higher than for colorblindness (M_multiculturalism_ = 5.52; SD_multiculturalism_ = 0.41; M_colorblindness_ = 5.39; SD_colorblindness_ = 0.40; *p* = 0.016). This response pattern is unusual, as multiculturalism and colorblindness are typically negatively correlated (Hachfeld et al., 2015; Kehl et al., 2024). It is unclear whether these teachers were considering different contexts when responding to the items, or if their responses reflect an attempt to give the “correct” answer without a clear understanding of what those might be. The smallest group, labeled “pro-colorblindness” (*N* = 40; 4% of the whole sample) included teachers who exhibited low endorsement of multiculturalism and high endorsement of colorblindness (M_multiculturalism_ = 1.92; SD_multiculturalism_ = 0.58; M_colorblindness_ = 4.47; SD_colorblindness_ = 0.90; *p* < 0.001). They generally believe that educators should ignore students’ backgrounds in teaching and learning.

To better understand the characteristics of the identified groups, we analyzed their gender, race/ethnicity, and education level. Table 1 displays the percentages of these demographic variables within each group, along with the overall sample percentages. The lean-multiculturalism group, representing a large portion of the sample, had a composition similar to the overall sample. The pro-multiculturalism group included a higher proportion of female teachers and teachers with Master’s degrees. The dual-belief group had slightly more female teachers, fewer White teachers, and a higher representation of Black/African American and Asian American teachers, as well as those with doctoral degrees. The pro-colorblindness group had more male teachers. In terms of race/ethnicity, it had lower proportions of Black/African American and Hispanic/Latino/American teachers, and higher proportions of White, Asian, and Native American teachers. Teachers holding 2-year associate degrees were underrepresented in this group relative to the overall sample, while those with doctoral degrees were overrepresented.

### 3.2. Individual Beliefs, Administrator Support, and Political Climate Jointly Predict Educators’ Implementation of Culturally Responsive Practices

We conducted a series of stepwise regression analyses to investigate the relationships between teachers’ personal diversity beliefs, administrator support, and political climate and their use of CRPs (see Table 2). Cases with mixed political climates (i.e., in states without anti-DEI legislation but located in precincts where the majority voted for the Republican candidate in 2020, as well as those in states with anti-DEI legislation but located in precincts where the majority did not vote for rump in 2020; N = 375) were excluded from the following regression analyses, as it is unclear how teachers would perceive the political climate when exposed to conflicting signals from their local community and state. We also excluded the dual-belief and pro-colorblindness groups because the sample sizes for these two groups were too small. Therefore, the analytical sample size for the regression analyses was 435. Although the analytical sample is significantly smaller than the overall sample, their demographic composition and descriptive statistics of the focal variables were similar. The comparison statistics can be found in Supplemental Materials Table S1. Due to the strong correlation between the two types of administrator support (r = 0.77, *p* < 0.001), we estimated separate regression models for district support and school support to avoid suppression effects (Pandey & Elliott, 2010; Smith et al., 1992).

**Figure 2:**
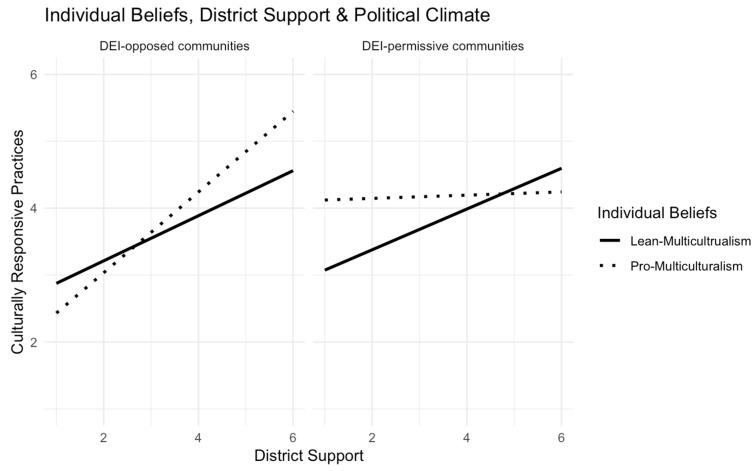
Interaction effects of teachers’ diversity beliefs, district support for equity, and political climate on the frequency of implementing CRPs.

**Figure 3:**
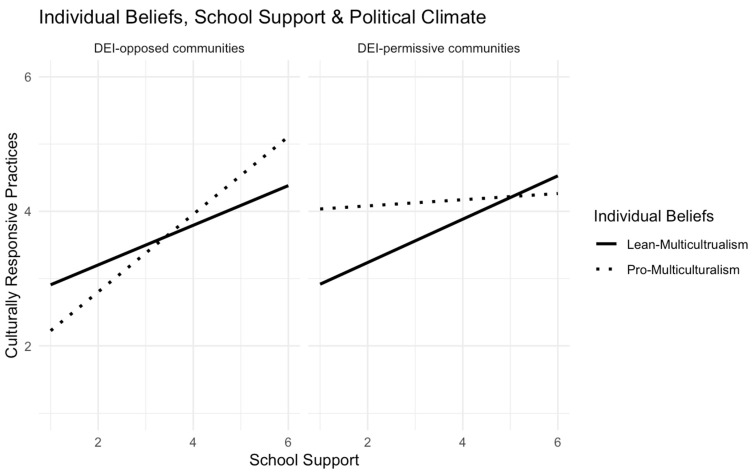
Interaction effects of teachers’ diversity beliefs, school support for equity, and political climate on the frequency of implementing CRPs.

**Model 1** included only teacher race and gender covariates. Neither was significantly associated with CRPs in this sample. **Models 2 and 3** introduced the main effects of teachers’ diversity beliefs, administrator support (district support in Model 2, and school support in Model 3), and political climate. **Models 4 and 5** tested the 3-way interactions among the focal variables. For both models, we observed significant 3-way interactions. As shown in **Figure 2**, teachers classified as “lean-multiculturalism” (solid line) demonstrated similar patterns across DEI-opposed and DEI-permissive communities. In both communities, these teachers reported implementing CRPs more frequently when they perceived greater district support for equity work (DEI-permissive communities: B = 0.27, *p* < 0.01; DEI-opposed communities: B = 0.37, *p* < 0.01). The “pro-multiculturalism” group (dashed line), however, demonstrated distinct patterns depending on the political climate. In DEI-permissive communities, these teachers implemented CRPs relatively often, regardless of perceived district support (B = 0.02, *p* = 0.84). However, in DEI-opposed communities, they implemented CRPs significantly less often when they perceived low district support (B = 0.64, *p* < 0.01). **Figure 3** illustrates the results of the 3-way interactions involving individual diversity beliefs, school support, and political climate. The results closely mirrored those observed with district support. Again, the “lean-multiculturalism” teachers (solid line) exhibited similar patterns across DEI-opposed and DEI-permissive communities. Their implementation of CRPs was positively associated with their perceived school support (DEI- permissive communities: B = 0.30, *p* < 0.01; DEI-opposed communities: B = 0.30, *p* < 0.01). In contrast, the “pro-multiculturalism” teachers (dashed line) showed varying patterns. In DEI-permissive communities, they implemented high levels of CRPs, regardless of perceived school support (B = 0.03, *p* = 0.74). However, in DEI-opposed communities, their use of CRPs was strongly influenced by their perceived school support (B = 0.58, *p* < 0.01).

## 4. Discussion

Concern for teachers’ use of practices that support educational equity has been called one of the most pressing issues in 21st-century education (Sleeter, 2011), particularly as the population of U.S. students continues to diversify. This study offers a unique examination of how individual and contextually derived moral frameworks about the role of diversity in education predict how teachers engage their increasingly culturally diverse students. Our findings align with prior research illustrating that individual moral frameworks about diversity (e.g., greater endorsement of multiculturalism, weaker endorsement of colorblindness) can predict increased use of CRPs (Comstock et al., 2023; Parkhouse et al., 2019), and that administrator support can play a pivotal role in supporting CRP implementation (Kelly et al., 2024). However, our findings also expand this literature by illustrating that these two factors are interconnected, and they carry different implications for teachers’ use of CRPs in communities that are more versus less opposed to DEI efforts in educational contexts.

When teachers who endorsed moral frameworks toward diversity that were rooted in multiculturalism (i.e., pro-multiculturalism teachers) worked in states that were not explicitly opposed to DEI, they implemented teaching practices that aligned with their individual moral frameworks (i.e., CRPs), regardless of the morality cues they perceived in their school contexts (i.e., administrators’ support for DEI efforts). However, when pro-multiculturalism teachers worked in communities that were opposed toward DEI, their implementation of CRPs depended upon the morality cues they perceived in their school contexts. Pro-multiculturalism teachers were less likely to implement CRPs when both school and political climate morality frameworks were opposed to these practices. Rather than suggesting that pro-multiculturalism teachers lack moral fortitude, we suspect that our findings reflect an effort by pro-multiculturalism teachers to align their teaching with their community’s values, or to protect themselves from the threats that all teachers face in states that are opposed to DEI (e.g., parental backlash and loss of employment as a consequence of implementing CRPs; Jayakumar & Kohli, 2023; Pollock, 2023). Without support from their administrators, even teachers with strongly codified multicultural diversity frameworks may be unwilling or unable to implement practices that align with these beliefs. With the recent political shifts at the federal level, it is likely that educators will -and may already- perceive an increasingly opposed political climate towards educational equity, which may generally undermine their willingness to implement practices like CRPs, even when they believe these practices to be effective. 

For teachers with less codified moral frameworks (i.e., lean-multiculturalism teachers), the overarching political climate exerted less influence on their use of CRPs. Instead, their practice implementation depended upon the morality cues embedded within their school contexts. When lean-multiculturalism teachers perceived their administrators as being supportive of educational equity, they were more likely to implement CRPs, regardless of how opposed the overarching political climate was to DEI. For these teachers, whose moral frameworks toward diversity may be less solidified or narrow compared to those of pro-multiculturalism teachers, administrator support may provide a moral framework that they can use to make decisions about their practices. Administrators’ explicit endorsement of efforts to promote educational equity can serve as a “roadmap” for these lean-multiculturalism teachers to align their teaching practices with school and district priorities. Without explicit contextual morality cues, lean-multiculturalism teachers may struggle or elect not to implement CRPs. For instance, Daniel and Pray (2017) found that teachers working in schools with conflicting language policies felt constrained in adopting CRPs, often reverting to traditional methods. Similarly, Leeman and van Koeven (2019) reported that teachers who recognized the benefits of multilingualism felt undermined by their schools’ monolingual policies, which made them feel powerless to challenge long-standing conventions by integrating multilingual instruction. These findings highlight the critical role administrators can play in shaping educators’ practice decisions. Teachers, especially those with less codified moral frameworks, such as the majority of our sample, look to administrators to make decisions about how to best engage students.

### Limitations and Future Directions

Our study utilized a large national sample of in-service K-12 teachers, which enabled us to explore the complex relationships between teachers’ moral frameworks toward diversity, administrator support, and the community political climate in relation to the implementation of culturally relevant practice. However, we recognize several limitations that should be addressed in future research.

First, the data collected from a single time point cannot demonstrate causality and should therefore be interpreted with caution. Moreover, the reliance on quantitative data prevented a satisfactory investigation of the distinct teacher groups identified by the profile analysis. For example, the emergence of the dual-belief group was both unexpected and intriguing. We do not have a clear understanding of why these teachers strongly endorse both multiculturalism and colorblindness. One possibility is that this dual belief group may intend to promote equity through their practices but lack knowledge of how colorblind practices impact culturally diverse students (Byrd, 2017). Alternatively, it is possible that strong meritocratic beliefs among these teachers might result in a high endorsement of colorblindness (in the form of meritocracy) alongside high multiculturalism (Bonilla-Silva, 2015). Researchers have demonstrated multiple typologies within colorblindness (i.e., emphases on merit, individuality, or sameness) and suggested typologies for multiculturalism (i.e., emphases on perspective taking or learning about cultural differences) that individuals differentially endorse by context (Knowles et al., 2009; Leslie & Flynn, 2022; Plaut et al., 2011). Future investigations should incorporate additional explanatory factors to clarify the distinctions between these rationales for diverse beliefs and explore qualitative methods to capture richer insights into their beliefs and practices.

Second, our measure of local support for DEI relied on political climate, which significantly reduced the analytical sample size in this study. We created a proxy measure for political climate based on participants’ state and zip code. While this measure provides a clear delineation of which states are opposed (vs. not) to DEI, we had to remove a substantial portion of data that lacked information regarding political climate. Future research should consider strategically sampling based on geographic locations to mitigate this issue and integrate subjective assessments of the political climate and local support for DEI.

Third, we assessed administrator support in a general way by asking teachers about their perceptions of how much their districts and schools prioritize equity work and make meaningful differences. Future studies could provide a more nuanced understanding of the role of administrator support in teachers’ decisions to implement CRPs, exploring the specific types of support administrators provide and evaluating their influence on teachers’ practice implementation decisions. For example, researchers could draw on prior research attending to mechanisms through which administrator support fosters teaching practices (Peurach et al., 2019). This would enable an analysis to provide more concrete administrator strategies for supporting equitable teaching practices.

Lastly, as this data set was collected in December 2023, it will be important to track how community political climate around DEI in K-12 continue to change. Voting returns from the most recent US presidential election indicated shifts in many communities. Additional research to understand the implications of the changing the political climate around DEI and its effect on schools, teachers, and schools will be essential.

## 5. Conclusions

This study highlights the interplay between individual and contextually derived moral frameworks toward diversity in influencing culturally responsive teaching practices and contributes to understanding how individual and contextual morality intersect to shape professional behavior in educational settings. By examining these dynamics across DEI-permissive and DEI-opposed communities, our findings point to the critical role that contextually derived moral frameworks play in either enabling or hindering teachers’ tendency to implement practices that align with their own moral frameworks. More than half of the teachers surveyed belong to the lean-multiculturalism group and rely heavily on the contextual morality cued by administrator guidance to implement CRPs. While a quarter of teachers with high multiculturalism beliefs relied on their more codified moral frameworks to guide their implementation of CRPs in communities that were not explicitly opposed to DEI, these teachers required the contextual moral support of administrators to implement the same practices in DEI-opposed communities. Our findings point to the key role that administrators play in helping teachers to make sense of the right or moral way to engage diverse students in communities with differing perspectives on DEI.

## Figures and Tables

**Table 1:** Demographic characteristics of the four identified teacher groups and the overall sample.

Demographic Characteristic		Lean-Multiculturalism (*N* = 697)	Pro-Multiculturalism (*N* = 278)	Dual-Belief (*N* = 82)	Pro-Colorblindness (*N* = 40)	Overall (*N* = 1097)
Gender ^1^	Male	21.7%	12.2%	15.9%	37.5%	19.4%
	Female	78.2%	86.7%	85.4%	62.5%	80.3%
Race ^2^	White	77.3%	78.4%	54.9%	87.5%	76.3%
	Black/African American	12.2%	14.4%	19.5%	0%	12.9%
	Hispanic/Latine American	10.8%	7.6%	14.6%	5%	10.0%
	Asian American	4.3%	3.2%	7.3%	7.5%	4.4%
	Native American	1.7%	2.2%	2.4%	7.5%	2.1%
Education	2-year degree (associate’s)	14.6%	7.2%	15.9%	5%	12.5%
	4-year degree (bachelor’s)	38.7%	33.8%	42.7%	42.5%	37.9%
	Master’s degree	34.7%	52.9%	22.0%	35%	38.4%
	Doctoral degree	3.3%	2.5%	8.5%	10%	3.7%

^1^ Three teachers identified as non-binary, and all of them were in Group 3, while one teacher identified as transgender and was in Group 4. ^2^ Four Middle Eastern/North African teachers were identified: two belonged to Group 1, one belonged to Group 3, and one belonged to Group 4.

**Table 2:** Results of Regression Analyses *(N* = 435).

	Model 1	Model 2	Model 3	Model 4	Model 5
Intercept	3.90 *** (0.12)	3.87 *** (0.13)	3.85 *** (0.13)	3.85 *** (0.14)	3.83 *** (0.14)
Race (0 = White; 1 = non-White)	0.12 (0.15)	0.09 (0.14)	0.10 (0.15)	0.04 (0.14)	0.07 (0.15)
Gender (0 = Male; 1 = non-Male)	0.21 (0.14)	0.16 (0.13)	0.17 (0.13)	0.15 (0.13)	0.16 (0.13)
Individual Belief (0 = lean-multiculturalism; 1 = pro-multiculturalism)		0.22 (0.12)	0.21 (0.12)	0.42 * (0.19)	0.33 (0.19)
District Support		0.30 *** (0.05)		0.37 *** (0.11)	
School Support			0.27 *** (0.06)		0.31 ** (0.10)
Political Climate (0 = DEI-opposed; 1 = DEI-permissive)		0.02 (0.11)	0.04 (0.11)	0.09 (0.13)	0.11 (0.13)
Individual Belief * District Support				0.29 (0.19)	
Individual Belief * Political Climate				−0.29 (0.24)	−0.20 (0.25)
District Support * Political Climate				−0.04 (0.14)	
Individual Belief * District Support * Political Climate				−0.60 * (0.23)	
Individual Belief * School Support					0.29 (0.19)
School Support * Political Climate					0.03 (0.14)
Individual Belief * School Support * Political Climate					−0.58 * (0.24)
Individual Belief * District Support * Political Climate				−0.60 * (0.23)	
Individual Belief * School Support					0.29 (0.19)

*** *p* < 0.001; ** *p* < 0.01; * *p* < 0.05.

## Data Availability

The data presented in this study are available on request from the corresponding author due to their use in ongoing research.

## Note

1 Within this paper, we conceptualize students from marginalized backgrounds as racial/ethnic minority students (e.g., students who are Black, Hispanic/Latine, or Native American) and students from lower-income families (Fluit et al., 2024).

## Supplementary Materials

The following supporting information can be downloaded at: https://www.mdpi.com/article/10.3390/bs15040446/s1, Table S1: Comparison of demographic characteristics and descriptive statistics between the overall sample and the analytical sample.
