# Supplementary material for: Individual and Contextual Morality: How Educators in Oppositional and Permissive Communities Use Culturally Responsive Practices
Source: Behav Sci (Basel). 2025 Mar 31;15(4):446. doi: 10.3390/bs15040446 (PMC12024385; doi:10.3390/bs15040446)
Supplement: Supplementary file 1 [file behavsci-15-00446-s001.zip › behavsci-3453427-supplementary.pdf]

# Supplementary Material

**Table S1.** Comparison of demographic characteristics and descriptive statistics between the overall sample and the analytical sample.

| | | Overall Sample | | Analytic Sample | |
|---|---|---|---|---|---|
| | | **Lean-multiculturalism (*N* = 697)** | **Pro-multiculturalism (*N* = 278)** | **Lean-multiculturalism (*N* = 301)** | **Pro-multiculturalism (*N* = 134)** |
| Gender | Male | 21.7% | 12.2% | 25.9% | 15.7% |
| | Female | 78.2% | 86.7% | 73.8% | 82.8% |
| Race | White | 77.3% | 78.4% | 82.7% | 82.1% |
| | Black/African American | 12.2% | 14.4% | 12.0% | 13.4% |
| | Hispanic/Latino/a American | 10.8% | 7.6% | 5.7% | 3.7% |
| | Asian | 4.3% | 3.2% | 5.3% | 5.2% |
| | Native American | 1.7% | 2.2% | 2.7% | 2.2% |
| Education | High School | 8.6% | 3.6% | 6.0% | 3.0% |
| | 2-year college degree (associate's degree) | 14.6% | 7.2% | 11.3% | 6.0% |
| | 4-year bachelor's degree | 38.7% | 33.8% | 41.2% | 31.3% |
| | Master's degree | 34.7% | 52.9% | 38.9% | 56.7% |
| | Doctoral degree | 3.3% | 2.5% | 2.7% | 3.0% |
| Focal variables | Multiculturalism | 4.36 (0.71) | 5.42 (0.44 | 4.35 (0.71) | 5.43 (0.46) |
| | Colorblindness | 3.48 (0.71) | 1.81 (0.47) | 3.43 (0.69) | 1.76 (0.47) |
| | Culturally Responsive Practices | 3.95 (1.18) | 4.23 (1.09) | 4.02 (1.20) | 4.25 (1.12) |
| | District Support | 4.23 (1.10) | 4.17 (1.29) | 4.25 (1.02) | 4.26 (1.24) |
| | School Support | 4.55 (0.96) | 4.55 (1.27) | 4.58 (0.96) | 4.60 (1.22) |
